# Targeting T Cell Metabolism for Improvement of Cancer Immunotherapy

**DOI:** 10.3389/fonc.2018.00237

**Published:** 2018-08-03

**Authors:** Thibault Le Bourgeois, Laura Strauss, Halil-Ibrahim Aksoylar, Saeed Daneshmandi, Pankaj Seth, Nikolaos Patsoukis, Vassiliki A. Boussiotis

**Affiliations:** ^1^Division of Hematology-Oncology, Beth Israel Deaconess Medical Center, Harvard Medical School, Boston, MA, United States; ^2^Department of Medicine, Beth Israel Deaconess Medical Center, Harvard Medical School, Boston, MA, United States; ^3^Division of Interdisciplinary Medicine and Biotechnology, Beth Israel Deaconess Medical Center, Harvard Medical School, Boston, MA, United States

**Keywords:** T lymphocytes, metabolism, immunotherapy, myeloid cells, cancer metabolism

## Abstract

There has been significant progress in utilizing our immune system against cancer, mainly by checkpoint blockade and T cell-mediated therapies. The field of cancer immunotherapy is growing rapidly but durable clinical benefits occur only in a small subset of responding patients. It is currently recognized that cancer creates a suppressive metabolic microenvironment, which contributes to ineffective immune function. Metabolism is a common cellular feature, and although there has been significant progress in understanding the detrimental role of metabolic changes of the tumor microenvironment (TEM) in immune cells, there is still much to be learned regarding unique targetable pathways. Elucidation of cancer and immune cell metabolic profiles is critical for identifying mechanisms that regulate metabolic reprogramming within the TEM. Metabolic targets that mediate immunosuppression and are fundamental in sustaining tumor growth can be exploited therapeutically for the development of approaches to increase the efficacy of immunotherapies. Here, we will highlight the importance of metabolism on the function of tumor-associated immune cells and will address the role of key metabolic determinants that might be targets of therapeutic intervention for improvement of tumor immunotherapies.

## Introduction

It is well-established that metabolic reprogramming is a hallmark of cancer progression (1–3). Compared to their normal cellular counterparts, malignant cells undergo major changes in metabolism to fulfill the biosynthetic and bioenergetic needs for rapid proliferation and adaptation to the stressful conditions of the tumor microenvironment (TME). Metabolic reprogramming and plasticity of cancer cells for such adaptations is considered a key mechanism of cancer treatment resistance (4). It is also well established that cancer progression is also intimately linked with the properties and function of immune cells in the TME. Several immune cell types, such as macrophages, B cells, T cells, NK and NKT cells, neutrophils, dendritic cells (DCs), and myeloid-derived suppressor cells (MDSCs), which are present in the TME, have an active role in the process of cancer progression (5, 6).

The metabolic state of the TME is regulated by the metabolic activity of the cancer cell, which alters the availability of nutrients in the microenvironment as a result of metabolic competition between cancer and immune cells for key nutrients, such as glucose, glutamine, lipids, and amino acids (7–9). The type of nutrients used by immune cells alters their differentiation program and functional properties. Changes in the availability of glucose, fatty acid, and amino acid guide the differentiation program of macrophages, DCs, and T cells (5, 10–16). Besides nutrient availability, high production of lactate, the end product of glycolysis, and the accumulation of multiple metabolic byproducts of cancer cell metabolism (17) are harmful for immune cells. As a consequence, differentiation of dendritic cell (DC) and macrophage is altered, and activation, fitness, and anti-tumor function of T cells are significantly impaired.

Metabolic changes related to TME hypoxia also affect the differentiation program of myeloid cells thereby altering their antigen-presenting properties (16, 18). Myeloid cells express ligands for multiple costimulatory and coinhibitory receptors present in T cells, which have a decisive cell-intrinsic role on the metabolic reprogramming and eventually the function of T cells in response to antigen encounter (19, 20). Hypoxia-mediated expression of HIF-1 in myeloid cells selectively upregulates the expression of inhibitory ligands, such as PD-L1, and promotes T cell immunosuppression (21). Such hypoxia-mediated changes also promote Treg differentiation and homeostasis (22), further suppressing the function of tumor-specific T effector cells.

Collectively, these studies strongly suggest that cancer-mediated metabolic changes in the TME impact the cellular composition and function of the immune microenvironment. Targeting metabolic changes of cancer cells will impact cancer cell growth and progression. Because such cancer cell-intrinsic metabolic changes affect the metabolism, differentiation, and function of tumor-infiltrating immune cells, metabolic vulnerabilities of cancer might be therapeutic targets for improvement of anti-tumor immunity by altering the metabolic program of immune cells and their anti-tumor function. Thus, mechanistic understanding of the metabolic imbalances in the TME might provide a means to develop novel therapeutic strategies to maximize the anti-tumor potential of the innate and adaptive immune system. As a consequence, such therapeutic targets could potentiate or alter the outcome of various types of immunotherapy, when combined. In the following sections, we will highlight the importance of metabolism on the function of tumor-associated immune cells and will address the role of key metabolic determinants that might be targets for therapeutic intervention for the improvement of tumor immunotherapies.

## Metabolism is a Key Feature of Every Cell

**Figure 1:**
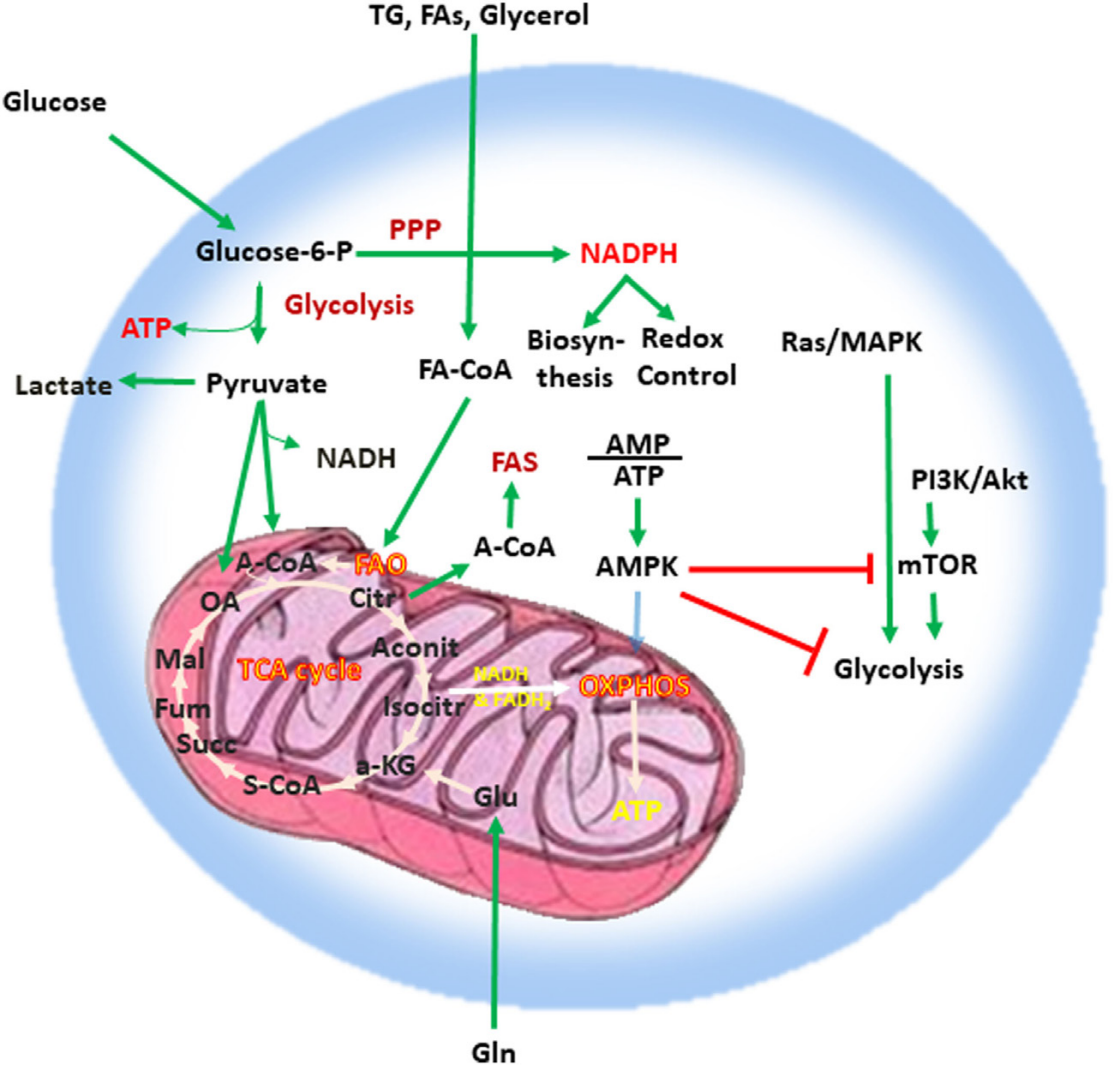
Metabolism is a key feature of every quiescent cell. Quiescent cells generate ATP by glycolysis and OXPHOS. Metabolism can be weighted toward anabolic reactions or toward catabolic reactions. Glucose is one of the main nutrients from which all types of cells generate energy. Glycolysis converts glucose into pyruvate *via* sequential enzymatic reactions, which lead to the generation of intermediate metabolites that can enter other pathways, such as the PPP. These coordinated metabolic processes are critical for successful biosynthesis and cell growth. Pyruvate generated from glycolysis can enter the mictochondria and can be converted into acetyl-CoA entering the TCA cycle or can be converted into lactate in the cytoplasm and excreted from the cell. Glycolysis also helps in the maintenance of the NAD^+^–NADH redox balance. Cells also use glutamine (Gln), which is metabolized by glutaminolysis, and lipids (TG, FA, and glycerol), which are metabolized by fatty acid oxidation. The intermediates produced by these catabolic processes enter the TCA cycle. The TCA cycle provides key substrates for biosynthesis, such as citrate, which can be exported to the cytosol and form the basis for FAS, whereas OXPHOS generates a high number of ATP thereby providing the high levels of energy required for cell growth. Abbreviations: α-KG, alpha-ketoglutarate; A-CoA, acetyl coenzyme A; Aconit, aconitase; Akt, protein kinase B; AMP, adenosine monophosphate; ATP, adenosine triphosphate; AMPK, AMP-activated protein kinase; Citr, citrate; FA, fatty acid; FA-CoA, fatty acyl coenzyme A; FAS, fatty acid synthesis; Fum, fumarate; Gln, glutamine; Glu, glutamate; Isocitr, isocitrate; Mal, malate; MAPK, mitogen-activated protein kinase; mTOR, mechanistic/mammalian target of rapamycin; NADH, nicotinamide adenine dinucleotide reduced; OA, oxaloacetate; OXPHOS, oxidative phosphorylation; PI3K, phosphatidylinositol-4,5-bisphosphate 3-kinase; PPP, pentose phosphate pathway; S-CoA, succinyl-coenzyme A; Succ, succinate; TCA cycle, tricarboxylic acid cycle; TG, triglyceride.

Adenosine triphosphate (ATP), the key energy-transporting molecule, is generated in every cell by glycolysis and oxidative phosphorylation (OXPHOS). Depending on the functional demands, cell metabolism can be shifted toward anabolic reactions leading to production of molecules involved in biosynthesis necessary for cell growth, or toward catabolic reactions leading to breakdown of macromolecules and the generation of products, which are subsequently used for energy production or for construction of anabolic pathways (3, 4, 23, 24). A balance of these anabolic and catabolic processes is mandatory for maintenance of metabolism homeostasis (Figure 1). Glucose is a main nutrient used by all cell types to generate energy during times of rapid growth, because using glucose for energy generation through glycolysis, spares other nutrients for usage in anabolic reactions. Moreover, glycolysis allows the rapid generation of metabolic intermediates, which can be used in other biosynthesic pathways necessary for cell growth. Glycolysis supports the pentose phosphate pathway (PPP) that has an important role in the production of building blocks necessary for nucleotide biosynthesis and generation of NADPH, which is mandatory not only for the support anabolic pathways but also for the redox state of the cell. Pyruvate derived from glucose in glycolysis can be converted into acetyl-CoA in the mitochondria entering the tricarboxylic acid (TCA) cycle or into lactate in the cytoplasm and excreted from the cell. Glycolysis also supports the redox balance of the cell through NAD^+^–NADH conversion.

Other critical nutrients include amino acids, as well as lipids, which can be metabolized *via* fatty acid oxidation (FAO) or used for biosynthetic reactions instead of energy production. The intermediates produced by catabolic reactions of amino acids and lipids also enter the TCA cycle. In addition to producing intermediates that feed multiple biosynthetic pathways, the oxidative reactions of the TCA cycle generate NADH and flavin adenine dinucleotide which are required for donation of electrons to the electron-transport chain for OXPHOS (Figure 1). OXPHOS is the energy power of the cell because of the abundant ATP production as it can generate 10 times more ATP molecules per molecule of glucose compared to glycolysis. Citrate is a key product of the TCA cycle, which forms the basis for fatty acid synthesis (FAS) after its export to the cytosol. In order to maintain functional integrity and ability to divide, a healthy cell must balance nutrient consumption and metabolism to successfully sustain energy, biosynthesis, and redox state.

## Metabolic Reprogramming of Cancer

**Figure 2:**
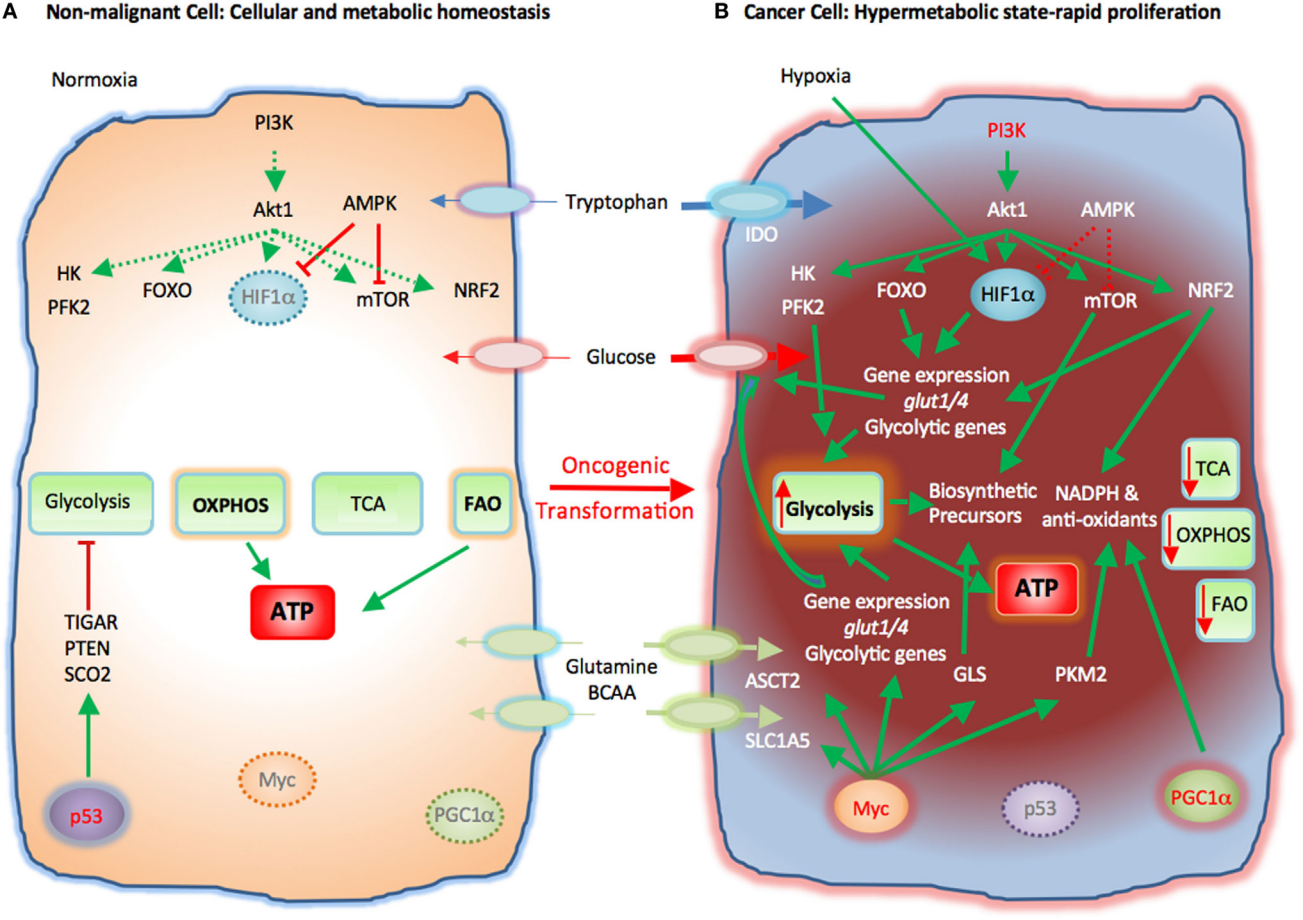
Metabolic reprogramming of cancer cells in the tumor microenvironment (TME). Metabolic switches driven by genetic alterations, alter the cell intrinsic properties of cancer cells leading to metabolic changes in the TME. **(A)** Nonmalignant cells have low level steady-state biosynthetic activity and low energy demands. Under normoxia, nonmalignant (quiescent) cells rely on oxidative phosphorylation (OXPHOS) as primary ATP source. Steady-state FAO also contributes to the cellular ATP pool. Without extrinsic stimuli the PI3K–Akt pathway is inactive and downstream targets, e.g., HK, PFK2, FOXO, HIF1α, mTOR, and NRF2, are not activated. Low levels of AMPK activity keep HIF1α and mTOR in check. p53 participates in the repression of glycolysis by expression of TIGAR, PTEN, and SCO2. Myc and PGC1α are not active in quiescent cells. **(B)** Cancer cells acquire mutations that promote glycolysis by multiple mechanisms. Oncogenic PI3K–Akt signaling and suppressed AMPK signaling induce activation of glycolytic enzymes such as HK and PFK2 and transcription factors such as FOXO. Hypoxia-induced HIF1α also promotes the expression of glucose transporters glucose transporter 1 (Glut1) and Glut4 and glycolytic enzymes. mTOR signaling is enhanced causing an increase in biosynthetic precursors. Activated PI3K–Akt signaling leads to upregulation of NRF2 and expression of glycolytic genes, NADPH, and anti-oxidants thereby protecting cancer cells from oxidative damage. PGC1α contributes to the intracellular anti-oxidant defense mechanisms. Mutation or deletion of p53 results in loss of glycolytic inhibitors, such as TIGAR, PTEN, and SCO2, whereas oncogenic Myc induces expression of glucose transporters and glycolytic genes resulting in dominance of glycolysis as the key metabolic pathway in cancer cells. Oncogenic Myc also promotes the expression of glutamine transporters and GLS. Myc also enhances the levels of cellular NAPDH and anti-oxidants *via* PKM2. Expression of IDO induces degradation of tryptophan to *N*-formylkynurenin. These molecular changes induce a dramatic augmentation of nucleotide, amino acid, and lipid biosynthesis, which are paired with enhanced catabolic pathways to enable cancer cells to proliferate rapidly. Abbreviations: Akt1, protein kinase B; AMPK, AMP-activated protein kinase; ASCT2, alanine, serine, and cysteine system amino acids transporter 2; ATP, adenosine triphosphate; BCAA, branched-chain amino acids; FAO, fatty acid oxidation; FOXO, forkhead-Box O; Glut1/4, glucose transporter1/4; HIF1α, hypoxia-inducible factor 1α; HK, hexokinase; IDO, indoleamine-pyrrole 2,3-dioxygenase; mTOR, mechanistic/mammalian target of rapamycin; Myc, Myc proto-oncogene; NADPH, nicotinamide adenine dinucleotide phosphate; NRF2, nuclear factor (erythroid-derived 2)-like 2; PFK2, phosphofructokinase 2; PGC1a, PPARg coactivator-1a; PI3K, phosphatidylinositol-4,5-bisphosphate 3-kinase; PTEN, phosphatase and tensin homolog; SCO2, cytochrome C oxidase assembly protein; TCA, tricarboxylic acid cycle; TIGAR, TP53 induced glycolysis regulatory phosphatase.

Rapid proliferation is a hallmark of cancer cells. To do so, cancer cells alter their energy metabolism from the metabolic pattern that dominates in their quiescent nonmalignant counterparts to a glycolytic program, which is the preferred form of energy metabolism even under aerobic conditions. This aerobic form of glycolysis is known as the Warburg effect (17, 23, 25). Tumor cells generate most of the required energy through uptake and utilization of glucose that is rapidly converted into lactic acid by glycolysis as opposed to mitochondrial OXPHOS, which is the main mechanism of glucose utilization in healthy quiescent cells (Figure 2). This glycolytic switch is useful not only for rapid generation of ATP but also for adaptation of malignant cells to the hypoxic TME (1). The metabolic shift of cancer cells to glycolysis is induced by various mechanisms (2, 5).

Cancer-induced mutations and alterations of signaling pathways activate PI3K-Akt, which promotes transcriptional induction of glucose transporters [e.g., glucose transporter 1 (GLUT1)], activation of glycolytic enzymes (e.g., HK2, PFKFB3), and parallel activation of mTOR. Activated mTOR induces expression or upregulation of the transcription factor hypoxia-inducible factor 1 (HIF1), which cooperates with other transcription factors or oncogenes, such as c-Myc, p53, or Oct1 to further upregulate the expression of glycolytic genes, including GLUT1, HK2, PFKFB3, LDHA, but also to suppress key enzymes of the TCA cycle, such as pyruvate dehydrogenase kinase (PDK), succinate dehydrogenase (SDH), or fumarate hydratase (26). These combined molecular and biochemical changes induce a metabolic reprogramming that almost uniformly results in glycolysis being the central mechanism of energy metabolism in cancer (17, 25).

Cancer cells require high consumption and utilization of glutamine, which supports their rapid replication (27). Through the process of glutaminolysis, glutamine is converted to glutamate by glutaminase (GLS) and subsequently to α-ketoglutarate (α-KG), which enters the TCA cycle and has a major role in amino acid, nucleotide, and FAS (Figure 2). Glutamine is also used to synthesize the key anti-oxidative metabolite glutathione, maintain cellular pool of NADPH, and maintain redox state (8, 28). To meet the increased demand for glutamine, cancer cells upregulate the glutamine transporter solute carrier family 1 member 5 (SLC1A5). Oncogenic Myc has an active role in the upregulation of SLC1A5 and ASCT2 (29) and also regulates the conversion of glutamine into a carbon source through glutaminolysis by upregulating the expression of GLS (25, 30, 31). Glutamine is also involved in protein translation because glutamine levels can regulate the function of mTORC1 (32). When sufficient amounts of glutamine and essential amino acids are present, activated PI3K–Akt or RSK activate mTORC1 (33). Under these conditions, a fraction of the imported glutamine is shuttled out of the cell in exchange for essential amino acids, which are utilized in mTORC1-mediated protein translation. Thus, glutamine regulates cancer cell metabolism and growth by multiple mechanisms as it serves as a direct precursor for protein synthesis but also regulates mTORC1 signaling and amino acid import thereby supporting protein translation (33).

In addition to glucose, glutamine and essential amino acids, cancer cells also alter their lipid metabolism leading to an anabolic program supporting lipogenesis. The enzyme fatty acid synthase (FASN) is highly expressed in cancer cells and its ablation inhibits cancer cell growth (1, 34, 35). Together, these key metabolic changes form the mechanistic basis of cancer progression (25).

The rapid proliferation of cancer leads to hypoxia, a key regulator of the TME features. Due to proliferation and lack of proportional vascular support, cancer cells quickly exhaust the available supplies of oxygen and create a hypoxic microenvironment (36). Under these conditions the growth advantage of cancer cells over nonmalignant cells depends on cancer cell adaptation to glycolysis and is driven by the transcription factor hypoxia-inducible factor 1α (HIF1α) which is stabilized by hypoxia (37). The oncogene-activated PI3K pathway, which is activated in many cancers, also stabilizes HIF1α even under normoxia (38, 39). HIF1α triggers transcriptional induction of glucose transporters and glycolytic genes (40) but, conversely, decreases pyruvate entry into the TCA cycle by promoting the transcription of pyruvate dehydrogenase kinases, thereby suppressing mitochondrial OXPHOS (41, 42). Importantly, oncogenic Myc collaborates with HIF1 to augment aerobic glycolysis whereas under physiologic conditions, HIF1 can inhibit Myc activity (43). High levels of Myc also activate the transcriptional expression of new target genes (44, 45).

## Metabolic Reprogramming of Macrophages

Macrophages have a central role in anti-tumor immunity by mediating direct anti-tumor functions and by regulating T cell immune responses. The classical polarization studies have identified that inflammatory stimuli such as interferon-γ (IFN-γ) together with LPS induce M1 macrophages, which produce inflammatory cytokines, such as interleukin-12, TNFα, IL-6, and IL-1, and generate reactive nitrogen and reactive oxygen intermediates (46). Conversely, anti-inflammatory factors, such as IL-4, IL-10, IL-13, and glucocorticoids induce differentiation of M2 macrophages which produce anti-inflammatory cytokines, and generate factors that induce immunosuppression, resolution of inflammation, and tissue remodeling. However, under natural in vivo immune responses, M1 vs. M2 phenotypes are rather a continuum instead of clearly distinct differentiation programs (47, 48).

To date most studies have suggested that M1 macrophages preferentially consume glucose, while M2 macrophages prefer the utilization of fatty acids. Consistently, M1 macrophages upregulate the glucose transporter Glut1 (49), while M2 macrophages increase expression of CD36 and lipoprotein lipase, which regulate the uptake and transport of fatty acids (50–52). However, recent studies have unraveled the complexity in fuel utilization, as they have identified enhanced consumption of glucose in M2 macrophages. Enhanced glucose consumption in M2 macrophages, sustains glycolysis as well as glucose oxidation, although the balance is shifted toward oxidation. This is in contrast to the metabolic preference of M1 macrophages, in which glycolysis dominates. Glucose uptake and catabolism is stimulated by Akt and interferon regulatory factor 4 (53) and regulates ATP citrate lyase to control metabolism-driven macrophage activation (54). Consistent with the complex programs of nutrient utilization, detailed comparative analysis of metabolic and molecular processes revealed a complex integration of metabolic and signaling pathways regardless of the type of macrophage polarization (55).

Since metabolism-driven changes in macrophages have a decisive role in their differentiation and function, metabolic changes of the TME are expected to alter macrophage differentiation. For example, glycolysis leads to accumulation of the TCA cycle intermediate, succinate, which by inducing the expression of HIF-1α can promote an inflammatory macrophage phenotype producing IL-1β (56). In contrast, itaconate functions as anti-inflammatory mediator in macrophages (57). Thus, depending on nutrient utilization and metabolite production, metabolism-driven differentiation of macrophages will be altered. Similarly, hypoxia-mediated expression of HIF-1α will also have a significant role in macrophage fate and function (56).

Studies during the past few years pinpoint cholesterol metabolism as a key regulator of macrophage function (58). It has been observed that in response to type I IFN signaling, macrophages increase cholesterol import but reduce cholesterol biosynthesis. This shift supports the expression of IFN-inducible genes and resistance to viral infection and is coordinated by STING. Because it resides at the endoplasmic reticulum (ER) where cholesterol is synthesized, STING may link sensing of cholesterol biosynthesis to type I IFN responses, thus defining a metabolic-inflammatory circuit that regulates antiviral defense (58).

## Metabolic Reprogramming of T Cells

Since the early era of immunotherapy, T cells have been acknowledged as central regulators of immune-mediated anti-tumor mediators (59, 60). Cytolytic CD8^+^ T lymphocytes (CTL) can mediate direct cytotoxic effects on tumor cells, whereas helper CD4^+^ T cells provide help for CTL function but also mediate direct cytotoxic activity.

T cells undergo metabolic reprogramming during activation which is critical for the acquisition of distinct differentiation profiles (61). Quiescent T cells produce energy through OXPHOS of various nutrients such as glucose and amino acids. During antigen encounter and activation, differentiating T effector cells have increased bioenergetic and anabolic needs to support rapid replication and production of soluble factors such as cytokines. To meet these needs, activated T cells increase the uptake of glucose and amino acids and their utilization by enhancing glycolysis, glutaminolysis, and catabolism of branched-chain amino acids (BCAA) (62). Activated T cells also increase the uptake of fatty acids but suppress FAO and promote lipid synthesis (63) (Figure 1). OXPHOS is also increased. In addition to enhanced glycolysis, glucose metabolism in the PPP is upregulated and together with glutaminolysis contributes to biosynthetic purposes, T cell effector functions, and fitness during the elevated metabolic and bioenergetics demands of the immune response (64–68). These metabolic changes are orchestrated by signaling pathways activated downstream of T cell receptor (TCR) and CD28 as well as by cytokine receptors, such as the PI3K–AKT–mTOR pathway which lead to the expression of transcription factors like HIF1α and c-Myc that regulate T cell metabolic programs and functional fates (62, 69). These signaling and molecular events induce glucose transporters such as Glut1, rate limiting enzymes of glycolysis such as HK2, and amino acid transporters, which together facilitate glycolysis and glutaminolysis (24, 70, 71). Importantly, many of these mechanistic changes induced in rapidly proliferating T cells highly resemble signaling and metabolic changes that dominate during cancer cell reprogramming.

The role of amino acids as key metabolic regulators of T cell differentiation and functional fate is well documented. Amino acids are key nutrients, because they can serve as source of fuel but also as precursors for synthesis of proteins and nucleic acids. TCR signaling increases the expression of the Slc7a5–Slc3a2 antiporter, also known as CD98, which imports BCAA, such as leucine, isoleucine, and valine, which activate mTORC1 and induce T cell metabolic reprogramming (72). TCR signaling also induces the expression of sodium-coupled neutral amino acids transporters SNAT1 (Slc1a5) and SNAT2 (Slc38a2) and the alanine, serine, and cysteine system amino acids transporter 2 (ASCT2) (70, 72, 73), all of which are capable of transporting glutamine. Glutamine, the most abundant amino acids in the blood, provides fuel for rapidly dividing T cells (62, 72). TCR-dependent uptake of glutamine and leucine is mediated by ASCT2 and results in activation of mTOR, differentiation of Th1, Th17 cells, and development of inflammatory T cell responses (73). Glutamine also has a mandatory role for CD8^+^ T effector cell fitness and development of CD8^+^ T memory (74).

Fatty acid metabolism has an important role in the differentiation of various T cell subsets. *De novo* FAS and fatty acid uptake are key features of T effector cells, whereas mobilization and utilization of stored esterified fatty acids synthesized from glucose is a feature of T memory cells (63). Importantly, *de novo* FAS vs. uptake control the differentiation decision between Th17 and Treg cells (75, 76). Berod et al. showed that inhibition of acetyl-CoA carboxylase 1 (ACC1) restrains the differentiation of Th17 cells and promotes the differentiation of anti-inflammatory Foxp3^+^ Treg cells. Th17, but not Treg cells, depend on ACC1-mediated de novo FAS and the underlying glycolytic-lipogenic metabolic pathway for their development. In contrast to Th17 cells which use this pathway to produce phospholipids, Treg cells uptake exogenous fatty acids for this purpose. These investigators found that pharmacologic inhibition or T cell-specific deletion of ACC1 not only blocked *de novo* FAS but also interfered with the metabolic flux of glucose-derived carbon *via* glycolysis and TCA cycle. These findings underline the fundamental differences between Treg and Th17 cells regarding the pathway selectivity for fatty acid sources (75). Importantly, the key regulator of T effector cell differentiation (77), mTOR, is also mandatory for Treg differentiation, function, and survival by inducing the expression of multiple genes with a key role on lipid metabolism (66). Furthermore, the transcription factor HIF1, a well-established regulator of glycolysis in cancer (40) and T effector cells (62, 78) is also required for Treg development and survival (22). Utilization of endogenous fatty acids is also a key mechanism for energy generation upon PD-1 ligation (20). Under these conditions, T cells are unable to uptake nutrients, such as glucose, glutamine, and BCAA but instead engage in FAO by mobilizing fatty acids from endogenous sources. It is possible that the degree of T cell exhaustion induced by PD-1 might depend on the reserves of endogenous lipids that can provide fuel for energy generation under conditions of engagement of this checkpoint inhibitor (19).

These extensive studies from multiple different systems reveal the complexity of metabolism-driven changes on the differentiation of various T cell subsets and indicate that therapeutic targeting of metabolic pathways may simultaneously alter T cell subsets with opposing functions.

## Immunometabolic Regulation of T Cell Responses in the TME is Governed by Crosstalk between Immune Cells and Cancer

### Metabolic Reprogramming of Cancer and Implications on T Cell Function in the TME

Cancer cells acquire unique biochemical properties to meet their demands for biosynthetic precursors and to minimize metabolic damage. These changes support growth programs, adaptation to various microenvironmental conditions with minimum damage, and survival under stress and/or limited nutrient availability. The cancer-specific molecular and biochemical programs allow nutrient utilization in a manner distinct from nonmalignant cellular counterparts. Such changes not only support cancer cell growth but also generate metabolic products, which alter the microenvironment and affect the fate and function of immune cells residing in proximity to cancer.

**Figure 3:**
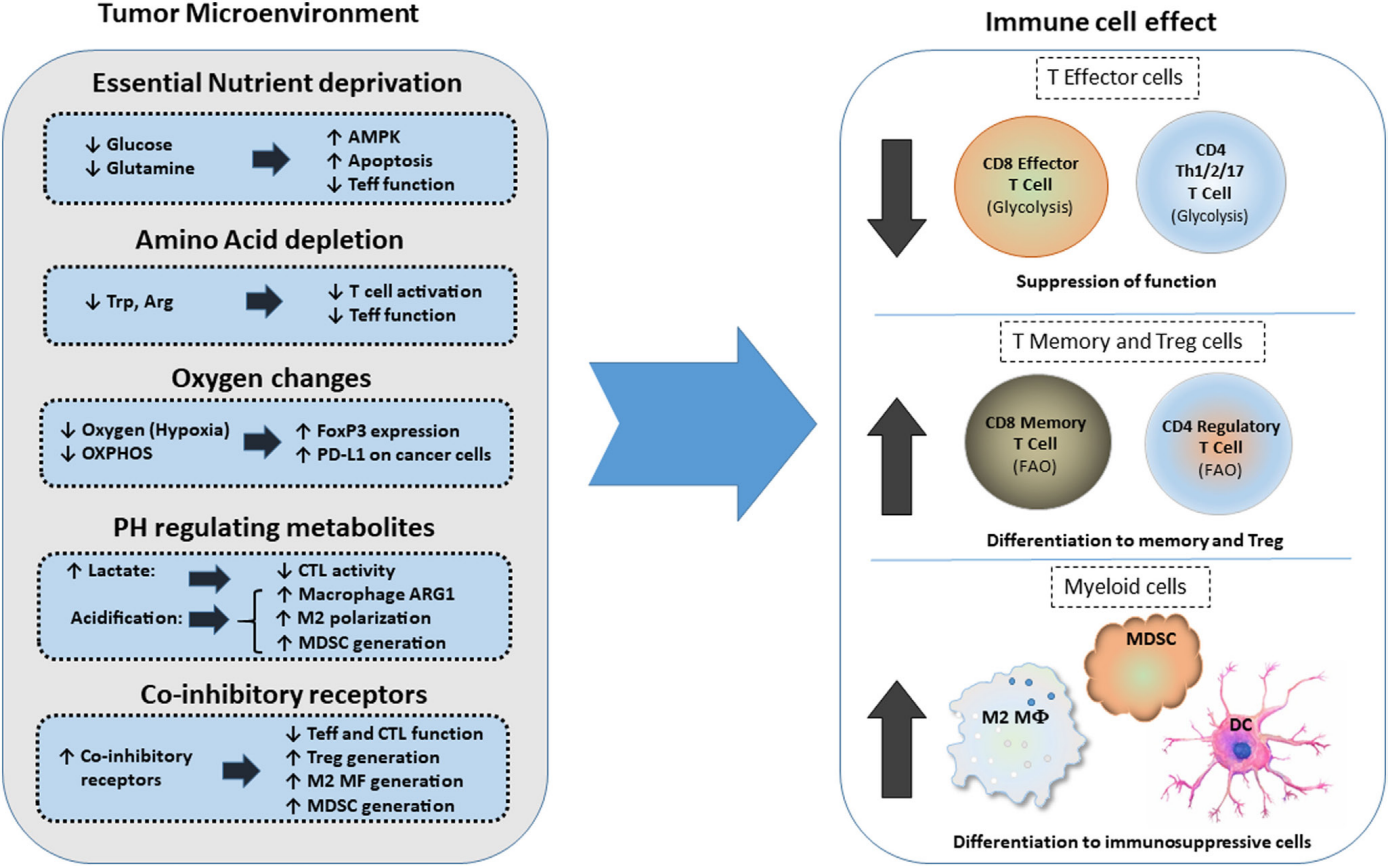
Effects of metabolic changes of the tumor microenvironment (TME) on immune cell differentiation. Metabolic changes of the TME driven by the increased metabolic activity of cancer cells alter the differentiation program of myeloid cells and T cells. In addition, expression of coinhibitory receptors alter signaling and cell-intrinsic metabolic reprogramming thereby limiting T cell cytolytic activity and promoting Treg differentiation and generation of suppressive myeloid cells. Abbreviations: AMPK, AMP-activated protein kinase; Arg, arginine; Arg1, arginase 1; CTL, cytolytic CD8^+^ T lymphocytes; DC, dendritic cell; FoxP3, forkhead-box P3; M2 MF, alternatively activated macrophage; MDSCs, myeloid-derived suppressor cells; OXPHOS, oxidative phosphorylation; PD-L1, programmed death-ligand 1; Teff, effector T cell; Th, T helper cells; Treg, regulatory T cells; Trp, tryptophan.

The high metabolic activity of cancer cells together with the poor vasculature blood supply in the TME can induce nutrient deprivation (Figure 3). These conditions of the TME can impair TCR signaling, glycolytic metabolism, amino acid uptake, and metabolism—all hallmarks of T effector cells—resulting in impaired anti-tumor effector functions of tumor-specific T cells. In contrast, Treg cells, which rely mainly on FAO (61, 67), can survive under these conditions and exert immunosuppressive effects on tumor-specific T effector cells. Expansion of Treg cells in the TME is also linked to the activation of AMPK, a sensor of nutrient deprivation and metabolic stress (74). Production of waste by the hypermetabolic cancer cells, such as lactate and metabolic products of amino acid metabolism like kynurenine, can inhibit T cell activation and cytolytic function and support Treg differentiation (5, 79). HIF1α, induced by TME hypoxia, can also promote the generation and maintenance of Treg cells (22). Hypoxia-induced HIF1α leads to the expression of PD-L1 in MDSC, thereby mediating potent immunosuppressive functions in tumor-specific T effector cells (21). Together the metabolic and nutrient changes that characterize the TME reshape metabolic reprogramming and have a decisive role on T cell differentiation by suppressing T effector cell differentiation and promoting multiple mechanisms of immunosuppression (Figure 3).

Coinhibitory pathways engaged in the TME can impact immune responses by altering T cell-intrinsic signaling and by modifying the metabolic properties and the function of innate immune cells (19). Not only the coinhibitory receptor ligands but also the coinhibitory receptors are present in various types of innate immune cells and might alter their metabolic properties and differentiation programs (21, 80–82). Intriguingly, the PD-1: PD-L1 axis is implicated in immunometabolic dysfunctions of monocytes in chronic lymphocytic leukemia (83). In that context, triggering PD-1 on monocytes hampers glycolysis and phagocytosis, whereas disrupting PD-1: PD-L1 signaling reverses these metabolic and functional defects.

PD-L1 expression on cancer cells is associated with cancer cell-intrinsic signaling *via* the PI3K/Akt pathway and mTOR, leading to upregulation of glycolysis genes and enhanced glycolysis (7). It is unclear whether PD-L1 can trigger reverse signals to cancer but it has been proposed that PD-L1 functions as a shield for cancer cells, protecting them from immune-mediated cell death and Fas-mediated killing (84). The anti-apoptotic effect of PD-L1 on cancer cells might result in simultaneous increase of PI3K/Akt activity and elevated rate of tumor-intrinsic glycolysis, both hallmarks of metabolically active, proliferating cancers. A recent study on melanoma and ovarian cancer cell lines either depleted (by shRNAs) or non-depleted of PD-L1 showed that tumor-intrinsic PD-L1 controlled tumor growth *in vitro* and *in vivo* (85). Significant gene expression differences were found in canonical and non-canonical autophagy pathways. *In vitro* and *in vivo* data from that study supported the role of PD-L1 in suppressing autophagy and in sensitizing tumor cells to autophagy inhibitors and showed that tumor PD-L1 expression predicts autophagy-dependent growth. These effects were mainly mediated through the mTOR pathway, supporting the concept, shown in previous studies in melanoma and sarcoma cells (7, 86), that tumor PD-1: PD-L1-dependent mTOR activity drives glycolysis and proliferation in cancer cells. Efforts to identify specific signaling motifs of the short intracytoplasmic sequence of PD-L1 revealed regulatory non-classical signal transduction motifs that counteract and confer resistance to IFN-β-mediated cytotoxic signals, protecting tumor cells from apoptosis by the STAT3–Caspase-7 axis (87).

Cancer-mediated metabolic alterations extend beyond the elevated needs of cancer cells for ATP production. Because, as a consequence of rapid proliferation, cancer cells generate reactive oxygen species (ROS), activation of mechanisms to sustain the balance of the intracellular redox level is a key component of metabolic adaptation. High levels of ROS create a toxic environment for T cells, which, unlike cancer cells, lack the cell intrinsic metabolic adaptations to survive under conditions of high ROS.

Together these metabolic changes of cancer cells have a major impact not only on cancer progression by supporting cancer cell growth but also generate metabolic products which alter the microenvironment and affect the fate and function of T cells residing in the microenvironment of cancer.

### Immunometabolic Responses of Innate Immune Cells in the TME

Two critical regulators of T cell activation and function in the TME are tumor associated macrophages (TAM) and the MDSC, which form two major innate cellular components. TAMs play a crucial role in cancer progression (88, 89). By producing reactive nitrogen species (RNS), ROS, and inflammatory cytokines, such as TNF, IL-1, and IL-6, TAMs contribute to cancer-mediated inflammation that leads to tumorigenesis (47, 88, 89). Moreover, by producing anti-inflammatory cytokines, such as cathepsins, metalloproteases, TGF-β, and IL-10, TAMs promote extracellular matrix remodeling, immunosuppression, cancer cell extravasation, and metastasis but also regulate response to chemotherapy (6, 90).

Myeloid-derived suppressor cells are defined functionally by the potent immunosuppressive effects that they exert on T cells (91). MDSCs comprise heterogeneous populations of early myeloid progenitor cells, including monocytic (M-MDSC) and granulocytic (PMN-MDSC) populations (48, 92). In mice, an initial characterization of M-MDSC and PMN-MDSC is provided by the CD11b^+^Ly6C^high^Ly6G^−^ and CD11b^+^Ly6G^+^Ly6C^low^ cell-surface markers, respectively. In humans, the equivalent M-MDSC and PMN-MDSC subsets are defined as CD11b^+^CD14^+^human leukocyte antigen-antigen D related^−/low^CD15 and CD11b^+^CD14^−^CD15^+^, respectively. The classic definition of MDSCs as immature myeloid cells that are blocked from differentiating has been recently challenged by studies which have suggested that M-MDSCs and PMN-MDSCs may represent differentiated monocytes and granulocytes that subsequently acquired immunosuppressive properties (93).

Amino acid metabolism and oxidative stress have important roles in mediating the suppressive function of MDSCs on tumor-infiltrating T cells (16). This is mediated by depletion of amino acids and by production of oxidative stress mediators such as ROS and NRS (48, 91). MDSCs deplete l-arginine through its metabolism *via* ARG1 and can sequester l-cysteine thereby depriving T cells from l-cysteine (94, 95). Depletion of these amino acids leads to inhibition of T cell proliferation. MDSC, DC, and TAM express indoleamine-pyrrole 2,3-dioxygenase (IDO), which catalyzes tryptophan metabolism in the kynurenine pathway (96, 97). IDO inhibits T cell activation by tryptophan deprivation and by promoting the expansion of Treg cells (98). By expressing NOS2, ARG1, and NADPH oxidase, the two major MDSC subsets induce the production of RNS such as nitric oxide (NO) and peroxynitrite, and ROS such as H_2_O_2_ (91). Monocytic MDSCs induce their inhibitory effect mainly *via* NO whereas granulocytic MDSCs *via* ROS. These ROS downregulate TCR and IL-2 receptor signaling, inhibiting T cell activation, expansion, and effector differentiation.

Alteration of lipid metabolism in the TME is associated with MDSC generation (16, 99). Hossain et al. showed that tumor-infiltrating MDSCs have increased fatty acid uptake and FAO (100). This was accompanied by upregulation of FAO enzymes, increased oxygen consumption rate (OCR), and increased mitochondrial mass. In that model, pharmacologic inhibition of FAO decreased the production of inhibitory cytokines and blocked the immunosuppressive functions of tumor-infiltrating MDSCs. FAO inhibition also delayed tumor growth and enhanced the antitumor efficacy of adoptive T cell therapy. Moreover, FAO inhibition, combined with low-dose chemotherapy, completely abrogated the immunosuppressive effects of MDSC and induced a significant antitumor T cell-mediated activity (100). In a recent study Al-Khami et al. showed that signaling through STAT3 and STAT5 by the tumor-derived cytokines, granulocyte colony-stimulating factor, and granulocyte-macrophage colony-stimulating factor (GM-CSF), induces expression of lipid transporters and increase the uptake of lipids, which are present at high concentrations in the TME (99). Intracellular accumulation of lipids enhances oxidative metabolism and promotes the immunosuppressive function of MDSC. Conversely, inhibition of STAT3 or STAT5 signaling or genetic deletion of the fatty acid translocase CD36 inhibits the activation of oxidative metabolism and prevents the immunosuppressive function of MDSC leading to enhanced CD8^+^ T cell functionality and delay in tumor growth. Moreover, human MDSC isolated from tumors and from peripheral blood also upregulate the expression of lipid transporters (101). In addition, incubation with lipids supports the generation of human MDSC with potent immunosuppressive function (99). These data strongly suggest that tumor-derived factors and the high lipid content of the TME can cause profound metabolic changes that govern the immunosuppressive function of MDSC.

In addition to lipids, glycolytic metabolites can modulate fitness, function, and differentiation of MDSCs and could be potential targets for anti-MDSC therapeutic strategy. When encountered with tumor-derived factors, myeloid cells upregulate glycolytic genes. Jian et al. observed that in response to GM-CSF, MDSCs exhibit higher glycolytic rate than their normal counterparts. In that system, upregulation of glycolysis prevented excess production of ROS by MDSCs and protected MDSCs from apoptosis. This effect was mediated by the glycolytic metabolite, phosphoenolpyruvate (PEP), which acted as a potent antioxidant (102).

Recently, MDSCs in the TME were found to overexpress HIF-1α, which was also required for their differentiation. An essential target of HIF-1α is PFKFB3, which induces the synthesis of fructose 2,6-bisphosphate, an allosteric stimulator of glycolysis and proliferation *via* stimulation of cyclin-dependent kinase-1. Grewal et al. recently reported that M-MDSCs induced by coculture with the melanoma cell line A375 express increased PFKFB3 and that exposure to the PFKFB3 inhibitor, PFK-158, reverses the suppressive function of these M-MDSCs on T cell activation. Furthermore, circulating MDSCs were markedly reduced in advanced cancer patients treated with PFKFB3 inhibitor (103). Therefore, selective inhibition of glycolytic intermediates, including PFKFB3, might be a novel therapeutic approach to target MDSCs. Thus, combinations of these inhibitors with immunotherapies might promote immune-mediated responses in cancer patients. This rationale, is further supported by the fact that hypoxia-induced HIF-1α is also involved in upregulation of PD-L1 in MDSC of the TME (21).

As reported for macrophages, a very recent study links cholesterol metabolism to MDSC expansion. Lei et al. found that the atorvastatin, which inhibits the rate limiting enzyme of cholesterol synthesis 3-hydroxy-3-methylglutaryl coenzyme A reductase (HMG-CoA reductase), promoted the expansion of MDSCs both *in vitro* and *in vivo* (104). Atorvastatin-derived MDSCs suppressed T cell responses and NO production seems to be actively involved in this immunosuppressive effect. Addition of the downstream metabolite of HMG-CoA reductase, mevalonate, almost abrogated the effect of atorvastatin on MDSCs, indicating that inhibition of the mevalonate pathway was involved in the atorvastatin-induced MDSC expansion (104). Statins, widely prescribed as cholesterol-lowering drugs, have been extensively studied for their pleiotropic effects on immune systems, due to the previously observed beneficial effects on autoimmune and inflammatory disorders (105, 106). However, these recent observations indicate that the mechanism of statin-induced immunosuppression has not been elucidated (107). While, as mentioned above, Lei et al. found that atorvastatin promoted the expansion of MDSCs (104), Ulivieri et al. reported that statins impair humoral and cell-mediated immunity and inhibit antigen cross-presentation and T cell activation (108). Thus, in cancer, statins might compromise anti-tumor immunity by various mechanisms. Further work is required to understand the role of these widely used drugs in the era of cancer immunotherapy.

### Immunometabolic T Cell Reprogramming in the TME

Metabolic reprogramming of T cells in the TME is regulated by direct effects on T cells and by crosstalk of T cells with innate immune cells and cancer (Figure 3). The coordinated metabolic switches in T cells modulate cellular activities and contribute to the progression of cancer. Metabolic crosstalk among T cells, innate immune cells, and cancer might govern immunometabolic regulations and impact anti-tumor responses of immune cells by regulating signals mediated by coinhibitory receptors and their ligands, which are expressed in cancer cells but also other cell types of the TME, including monocytes, macrophages, and stroma (109).

Immunometabolic regulations mediated by coinhibitory receptors can impact T cell responses due to direct effects on T cell-intrinsic signaling (19). When the TCR is engaged, tyrosine phosphorylated CD3 chains recruit kinases and scaffold proteins and promote activation of signaling cascades, generation of second messengers, and initiation of transcriptional events, which lead to T cell differentiation. These signaling pathways synergistically promote glycolysis and anabolic metabolism to support not only clonal expansion but also differentiation of CD4^+^ and CD8^+^ T cells (71, 110, 111). Metabolic mediators function as intermediates between the signaling events and the outcomes of T cell activation (19). Costimulatory receptors have a major impact on T cell differentiation by regulating metabolic programs during T cell activation (20, 71).

Several costimulatory and coinhibitory receptors and their ligands are indispensable for the induction and maintenance of T cell tolerance. These pathways include the B7–CD28, TIM, CD226–TIGIT–CD96 families, as well as lymphocyte activation gene 3, and the TNF receptor superfamily (112–114). Coinhibitory receptors provide a balance on the activation and expansion of antigen-specific T cells upon encounter with antigen and promote the differentiation and function of Treg (115, 116). Through these two mechanisms the coinhibitory receptors function as key regulators of self-tolerance and mandatory safeguards for prevention of autoimmunity. Ligands for coinhibitory receptors are expressed on various types of antigen-presenting cells (APCs). Importantly, cancer cells also express ligands for coinhibitory receptors and by doing so, exploit these potent mediators of natural tolerance to evade immune surveillance (109, 117).

Coinhibitory receptors have a major impact on the T cell differentiation and proliferation. Importantly, these two endpoints are regulated by T cell metabolism (61, 118). Since the various coinhibitory receptors differentially affect activation of signaling pathways, their role on altering the metabolic programs of T cells is also anticipated to be distinct. Thus, targeting immunometabolic pathways regulated by distinct coinhibitory receptors might have significant clinical implications by promoting the desired modifications in the metabolic programs that fuel T cell functional fate.

Dysregulated metabolism also contributes to TIL exhaustion in the TME. Hypoxia and hypoglycemia, two major metabolic challenges within the TME, impair CD8^+^ TILs through distinct mechanisms. Zhang et al. determined that CD8^+^ TILs experiencing double metabolic jeopardy enhance PPARα signaling and FA catabolism, as a last resource to preserve energy production. Supporting this metabolic program by the pharmacologic regulator of FA catabolism, fenofibrate, prolongs functionality of these exhausted CD8^+^ T cells, and delays tumor growth (119).

## Therapeutic Implications: Integrating Metabolism and Immunotherapy

A major goal of modern immunotherapy is the generation of novel approached to generate tumor-specific T effector cells with enhanced function, in parallel to the generation of T memory cells with enhanced viability and plasticity for effector differentiation upon re-exposure to cancer antigens. This will allow for long-lasting immune-mediated anti-tumor function instead of a transient anti-tumor effect. Because metabolism drives T cell differentiation, combining metabolism-targeting drugs with checkpoint inhibitors forms an attractive therapeutic idea that might alter the differentiation of tumor-specific T cells to promote the generation of potent T effectors and long-living memory cells and prevent the accumulation of exhausted T cells.

As outlined above, metabolic changes alter the phenotype and function of immune cells in the TME. During the tumor onset, glycolytic metabolism in TAMs would induce production of inflammatory cytokines, RNS, and ROS, which support cancer-related inflammation and oncogenic transformation. Subsequently, as cancer progresses, nutrient deprivation and accumulation of cancer-generates metabolites such as lactate can induce an immunosuppressive phenotype in TAMs and DCs. ARG1 and IDO produced by TAM, DC, and MDSCs also induce amino acid deprivation in the TME and compromise T effector differentiation. These events combined, inhibit anti-tumor T effector cell responses while inducing Treg generation and eventually promote tumor progression (Figure 3).

**Figure 4:**
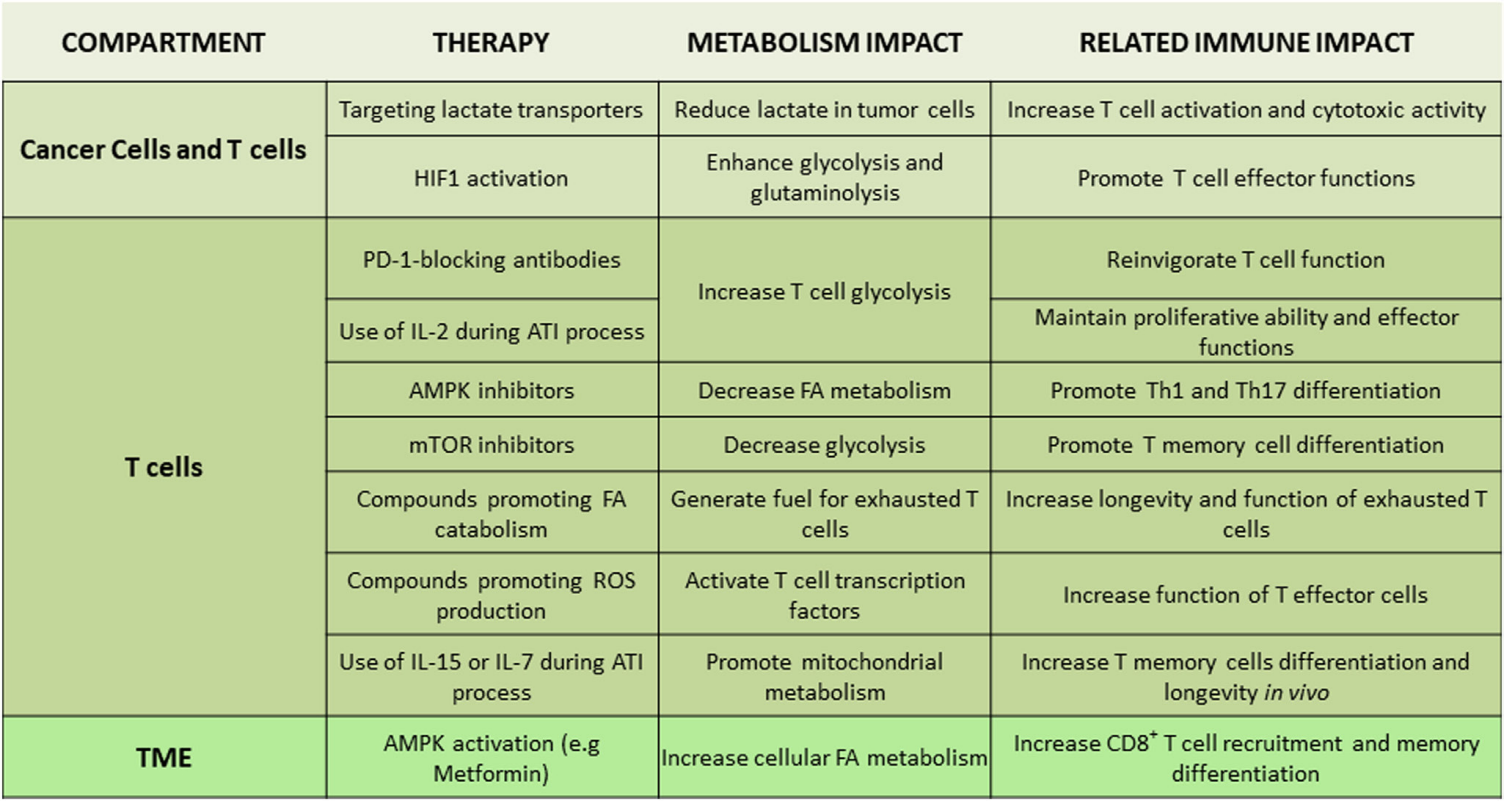
Tentative therapeutic targets for integration of metabolism and immunotherapy. Non-exhaustive representation of potential therapies to integrate metabolism in immunotherapy, description of the metabolism impact, and the related immune impact for each targeted therapy. Abbreviations: AMPK, AMP-activated protein kinase; ATI, adoptive T cell immunotherapy; FA, fatty acid; HIF1, hypoxia-inducible factor-1; IL-2/7/15, interleukin-2/7/15; Th, T helper cells; TME, tumor microenvironment.

Monocarboxylate transporters (MCTs) are family of transmembrane proteins which, include MCT1, MCT2, MCT3, and MCT4 that mediate proton-linked bidirectional movement of lactate and other metabolites such as ketone bodies and branched-chain ketoacids (120). MCTs control intracellular lactate and pH and have an important role for survival of cancer cells by preventing toxicity related to their hypermetabolic state. MCT1 and MCT2 are predominantly involved in the uptake of catabolites, such as lactate used in reverse Warburg pathway, and are highly expressed in certain types of cancer, which display rapid growth (121). Importantly, it has been reported that uptake of ketone bodies and lactate mediated by MCT1 and MCT2 feed mitochondrial metabolism preferentially in cancer stem cells (122). In that setting, a specific MCT1/2 inhibitor prevented the uptake of these metabolites and significantly inhibited growth and sphere formation of ER-positive and ER-negative breast cancer. Because accumulation of metabolic products and TME acidification affects the properties of immune cells, MCT-mediated function will have direct implications in immune cells of the TME (Figure 4). Indeed, MCT1-mediated export of branched-chain ketoacids by glioblastoma reduced the phagocytic activity of TAMs (123). The therapeutic potential of MCTs targeting is currently being tested in clinical trials with promising results generated by the MCT1 inhibitors SR12800 and AZD3965 (124, 125) and the dual MCT1/MCT2 inhibitor AR-C155858 (126).

Due to the intimate link between TME metabolic profile and T cell immune responses, various metabolites and metabolism-regulating molecules, such as lactate, HIF1, c-Myc, AMPK, and mTOR, are being tested as candidate therapeutic targets (Figure 4). Regulators of AMPK activity such as metformin or 5-aminoimidazole-4-carboxamide ribonucleotide have been evaluated for anti-tumor effects in preclinical models and in clinical trials (127, 128). AMPK might be an attractive target due to its effects in cancer but also T cells. By activating AMPK, metformin has a direct effect on immune cells leading to increased differentiation of CD8^+^ memory T cells (129) and possibly protection from apoptosis leading to improved outcomes of cancer vaccines (130). Additionally, AMPK has an important role for metabolic adaptation of T cells under conditions of stress and is required for metabolic fitness of effector T cells (74). However, AMPK activation can also promote the formation of Treg while reducing Th1 and Th17 cells (67), leading to an unwanted immune modulation in the context of cancer. Decrease of Th1 cells is expected to have detrimental effects on anti-tumor function (131), whereas compromising Th17 differentiation might decrease the longevity and anti-tumor potency of tumor-specific T cells (132). Furthermore, although metformin has been identified as an activator of AMPK, it has also been found to have other functions. Metformin can mediate direct inhibitory effects on glycolysis of cancer cells by inhibiting the rate limiting enzyme HK2 (133) but also has direct effects on the mitochondrial electron transport chain by abrogating the function of complex I (134). Thus, the net outcome of AMPK targeting on systemic anti-tumor immunity might vary among different cancers as it will depend on the properties of cancer and the type of immune cells that dominate the TME in each cancer type.

An attractive metabolic target is mTOR, which is activated both in cancer and immune cells. Targeting mTOR in cancer will promote apoptosis and nutrient deprivation (135, 136), whereas inhibition of mTOR in T cells can promote the differentiation of memory T cells (137). However, administration of mTOR inhibitors can also affect the differentiation of T effector cells, Tregs, and macrophages, all of which appear to utilize this key metabolic regulator for their differentiation and function (66, 77, 138). As a consequence, the outcomes of mTOR inhibition in cancer models are discordant and possibly dependent on the immune cell populations that are dominant in each experimental model.

Manipulating the cellular fatty acid metabolism might also be of therapeutic interest. Any modifications in basic cellular lipid metabolism can significantly affect T cell fate and function (76). The activation-induced proliferation and differentiation of effector T cells is supported by FAS, whereas the development of CD8^+^ T cell memory cells requires FAO (63). However, FAO is also important for the differentiation of CD4^+^ Treg cells (67) and its blockade could prevent the accumulation of this immunosuppressive population. Similarly, FAO is utilized by MDSC and has a critical role in MDSC-mediated T cell suppressive function (99, 100). Thus, therapeutic targeting fatty acid metabolism *in vivo* will affect more than one immune cell populations and might have unpredictable outcomes on the systemic antitumor effects. Alternatively, enhancing T cell fatty acid metabolism might be a therapeutic option in conditions of tumor-mediated T cell exhaustion when T cells depend only on FAO as the source of energy generation (20). In fact, Zhang et al. showed that in tumor-bearing mice, pharmacologic induction of fatty acid catabolism by fenofibrate prolongs functionality of exhausted CD8^+^ T cells, which cannot use other nutrients for energy generation in the hostile TME, and delays tumor growth when used together with PD-1-blocking immunotherapy (119).

As mentioned above, the function of mitochondria, which are the powerhouse of the cell, is suppressed by the effects of coinhibitory receptors, particularly PD-1 (19, 20). ROS, which are important mediators of T cell activation and function, are generated at complexes I, II, and III of the mitochondrial electron transport chain and have a key role in the function of innate and adaptive immune cells (139). Although high ROS levels are harmful (140, 141), ROS also function as signaling messengers in a multitude of pathways and superoxide converted from production of ROS activates CD4^+^ and CD8^+^ T cells by mediating transactivation of NFAT, NF-kB, and AP-1, and secretion of IL-2 (139, 142, 143). In a mouse tumor model, Chamoto et al. showed that the use of pharmacologic compounds that enhance ROS, such as ROS precursors or mitochondrial uncouplers can synergize with PD-1 blocking immunotherapy leading to improved anti-tumor responses (144). This combined treatment approach resulted in expansion of T effector and effector-memory cytotoxic cells in the tumor and the tumor-draining lymph nodes. These cytotoxic cells displayed enhanced activation of mTOR and AMPK. Although these results are promising, further investigation is required in order to allow clinical translation of these observations. For example, human peripheral blood mononuclear cells stimulated with a ROS generator developed Th2 and inhibited Th1 differentiation (145). Moreover, the use of mitochondria-targeting compounds may have severe toxicity in vital organs which are sensitive to oxidative stress (146–148). Thus, to employ such approaches for therapy in patients, development of successful strategies for precise drug delivery to specific cell types is first required.

Adoptive T cell immunotherapy (ATI) is a cancer treatment approach in which T cells from a patient are genetically engineered *in vitro* expanded by various methods and are subsequently reinfused in the patient as a therapeutic approach for targeted killing of cancers. To achieve successful cancer lysis *in vivo*, T cells generated for ATI should have proliferative ability and effector function. However, such cells should also be resistant to activation-induced cell death (AICD) and have the ability to convert to long-lasting T memory cells that will be able to remain quiescent but also re-gain effector function in order to attack potentially relapsing cancer. Several approaches have been tested to achieve the properties required for the generation of a T cell population that meets the requirements of optimal function after adoptive transfer by exploiting the function of costimulatory receptors and cytokines (149). Because effector and memory T cell differentiation and function are regulated by metabolism-driven processes, manipulating T cell metabolism is an attractive approach to enhance immunity or promote T cell survival and longevity for ATI. Enhanced glycolysis can promote T effector cell generation but also terminal differentiation, while inhibition of glycolysis leads to the generation of CD8^+^ T cells that have memory cell-like properties and maintain superior antitumor function and longevity (150, 151). Culturing human T cells destined for ATI in the presence of IL-2 might enforce T effector cell generation because IL-2 strongly promotes glycolysis (152). Although IL-2 has been historically considered as a pro-survival factor for dividing T cells, the enhanced activation induced in the presence of TCR-mediated signals and IL-2, might also drive terminal differentiation of T effector cells or promote AICD. In addition to undergoing AICD, T cells that are addicted to glycolysis during *in vitro* culture will suffer nutrient deprivation when entering the host and will die due to lack of sufficient glucose supplies. In contrast, IL-15 or IL-7 that promote memory cell differentiation (152) might promote longevity *in vivo*. However, a major challenge remains the need to achieve the T cell plasticity required for successful and long-lasting therapeutic outcome of ATI. For rapid therapeutic effect, these *ex vivo* engineered T cells should have the ability to mediate immediate anti-tumor function but also convert to memory T cells that remain viable in the host and are able to re-gain effector function if tumor relapses.

Recent studies have indicated that highly effective anti-tumor function is mediated by T cells which express a “hybrid” immunological and functional Th1/Th17 phenotype (153). Th1 is associated with enhanced effector function (154), whereas Th17 is associated with stemness and longevity (132, 155, 156). Using two different melanoma mouse models, Chatterjee et al. found that hybrid T cells with combined properties of Th1 and Th17 had the ability to mediate potent anti-tumor effector function but also displayed prolonged survival and persistence in vivo thereby mediating a sustained anti-tumor effect. These properties of Th1/Th17 hybrid cells were dependent on the increased NAD levels and the elevated activity of the histone deacetylase Sirt1, which is dependent on NAD. The causative role of this pathway in the function of the hybrid Th1/Th17 cells was established by genetic or pharmacologic ablation of Sirt1 activity, which compromised the antitumor function of Th1/Th17 cells. Conversely, deceased expression of CD38 NADase, which resulted in elevated levels of NAD, induced a dramatic anti-tumor effect (153). These observations provide the exciting potential that pharmacologic intervention to induce generation of such Th1/Th17 hybrid T cells might represent a highly promising approach for improvement of ATI.

The important functional role of metabolic reprogramming and its potential for therapeutic exploitation in ATI is supported by studies in chimeric antigen receptor (CAR)-T cells, a form of ATI that has revolutionized therapy in B cell malignancies. CARs are synthetic molecules that integrate the co-stimulatory domains of T cells with the specificity of antibody-binding domains. CAR T cells with 4-1BB costimulatory domains (157) appear superior to those that with CD28 costimulatory domains (158). The new generation CARs with additional costimulatory domains, such as CD28, 4-1BB (CD137), OX40, and inducible T-cell costimulator (159, 160) elicit potent T cell antitumor effects. These were designed to overcome anergy observed in first-generation CARs generated with CD3z signaling modules alone. Not only these modifications photocopy key features of natural co-stimulation such as enhanced proliferation, survival, and effector function of CAR T cells (157, 161) but also ameliorate exhaustion (162).

A recent study of these second-generation CARs showed a significant alteration in the differentiation and reprogramming of metabolic profiles of CAR T cells using CD28 or 4-1BB signaling domains. CAR signaling domains reprogram T cell metabolism resulting in preferential utilization of aerobic glycolysis in the 28ζ CAR T cells, whereas 4-1BBζ CAR T cells, oxidative breakdown of fatty acids was significantly enhanced. Moreover, 4-1BBζ CAR T cells generated increased SRC compared to 28ζ CAR T cells. This was accompanied by increased expression of genes that modulate transcriptional networks of mitochondrial biogenesis and oxidative metabolism in 4-1BBζ CAR T cells (163). Because T memory cells display elevated basal OCR and spare respiratory capacity (SRC), the enhanced oxidative features observed in 4-1BBζ CAR T cells might indicate increased reliance on FAO (164). Indeed, the 4-1BBζ signaling domain leads to increased frequency of central memory T cells, whereas 28z promotes to an effector memory differentiation population (163). Since SRC enhances survival and function of memory T cells by providing an exigency energy source (165), it is likely that these features may be necessary for central memory differentiation and survival of CAR T cells in hypoxic and nutritionally deprived TME resulting in better therapeutic outcome compared to first-generation CARs. The distinct metabolic programs induced by 4-1BBζ vs. CD28ζ CART are consistent with previous reports implicating 4-1BB signaling in long-term survival benefits to T cells (166) and signaling pathways used by 4-1BB are distinct from CD28 (167).

In conclusion, the function of every cell present in the TME is supported by metabolism. Immunometabolic pathways provide a key determinant of the functional fate of myeloid cells and T cells and control their qualitative, quantitative, and fitness program ultimately regulating anti-tumor immunity. As a consequence, mechanistic understanding of such immunometabolic changes provides the means for the development of novel therapeutic targets to improve T cell immune function. Identifying metabolic pathways that are shared between cancer and immune cells will allow the selection of metabolism-targeting drugs previously developed for the treatment of cancer, as candidate immunomodulators by reprogramming T cell metabolism. Using such drugs together with chemotherapy, antibody-based immunotherapy, ATI, and cancer vaccines may open new opportunities in improving cancer therapy.

## Author Contributions

TB wrote manuscript and generated figures. LS wrote sections of the manuscript. H-IA wrote sections of the manuscript and generated abbreviation list. SD wrote sections of the manuscript. PS wrote sections of the manuscript. NP wrote sections of the manuscript and generated figures. VB wrote sections of the manuscript, generated figures, and organized the contributions of the individual authors. All authors read and edited the complete manuscript.

## Conflict of Interest Statement

The authors declare that the research was conducted in the absence of any commercial or financial relationships that could be construed as a potential conflict of interest.

## Appendix

### Abbreviations

4-1BB - tumor necrosis factor receptor superfamily member 9

α-KG - alpha-ketoglutarate

ACC1 - acetyl-CoA carboxylase 1

acetyl - CoA - acetyl coenzyme A

Acly - ATP citrate lyase

AICAR - 5-aminoimidazole-4-carboxamide ribonucleotide

AICD - activation-induced cell death

AKT - protein kinase B

AMP - adenosine monophosphate

AMPK - AMP-activated protein kinase

AP-1 - adaptin1, activator protein 1

APC - antigen-presenting cells

ARG1 - arginase 1

ASCT2 - alanine, serine, and cysteine system amino acids transporter 2

ATI - adoptive T cell immunotherapy

ATP - adenosine triphosphate

BCAA - branched chain amino acids

CAR - chimeric antigen receptor

caspase - cysteine-aspartic protease

CDK1 - cyclin dependent kinase-1

CTL - cytolytic CD8+ T lymphocytes

DC - dendritic cell

ER - endoplasmic reticulum

FADH2 - flavin adenine dinucleotide

FAO - fatty acid oxidation

FAS - fatty acid synthesis

FASN - fatty acid synthase

FOXO - forkhead-box O

FOXP3 - forkhead-box P3

FH - fumarate hydratase

Gln - glutamine

GLS - glutaminase

G-CSF - granulocyte colony-stimulating factor

GM-CSF - granulocyte-macrophage colony-stimulating factor

GLUT1 - glucose transporter 1

HIF-1α - hypoxia-inducible factor 1α

HK2 - hexokinase 2

HLA-DR - human leukocyte antigen-antigen D related

HMG-CoA reductase - 3-hydroxy-3-methylglutaryl coenzyme A reductase

ICOS - inducible T-cell costimulator

IDO - indoleamine-pyrrole 2,3-dioxygenase

IFN - interferon

IFNγ - interferon γ

IL-1/2/4/6/7/10/13/15 - interleukin 1/2/4/6/7/10/13/15

IRF4 - interferon regulatory factor 4

LAG-3 - lymphocyte activation gene 3

LDHA - lactate dehydrogenase

M1 - classically activated macrophage

M2 - alternatively activated macrophage

MAPK - mitogen-activated protein kinase

MCT - monocarboxylate transporter

MDSCs - myeloid-derived suppressor cells

mTOR - mechanistic/mammalian target of rapamycin

mTORC1 - mechanistic/mammalian target of rapamycin complex 1

Myc - Myc proto-oncogene

NAD - nicotinamide adenine dinucleotide

NADH - nicotinamide adenine dinucleotide reduced

NADPH - nicotinamide adenine dinucleotide phosphate

NFAT - nuclear factor of activated T cells

NF-kB - nuclear factor ‘kappa-light-chain-enhancer’ of activated B-cells

NK - natural killer cells

NKT - natural killer T cells

NO – nitric oxide

NOS2 - nitric oxide synthase 2

OCR - oxygen consumption rate

OXPHOS - oxidative phosphorylation

PD-1 - programmed death-1

PD-L1/2 - programmed death-ligand 1/2

PDK - pyruvate dehydrogenase kinase

PEP - phosphoenolpyruvate

PFK2 - phosphofructokinase 2

PFKFB3 - 6-phosphofructo-2-kinase/fructose-2,6-biphosphatase 3

PGC-1a - PPARγ coactivator-1a

PI3K - phosphatidylinositol-4,5-bisphosphate 3-kinase

PKM2 - pyruvate kinase M2

PMN - polymorphonuclear neutrophil/leukocyte

PPARα - peroxisome proliferator-activated receptor α

PPARγ - peroxisome proliferator-activated receptor γ

PPP - pentose phosphate pathway

PTEN - phosphatase and tensin homologue deleted on chromosome 10

RNS - reactive nitrogen species

ROS - reactive oxygen species

RSK - ribosomal protein S6 kinase

SCO2 - cytochrome C oxidase assembly protein

SDH - succinate dehydrogenase

shRNA - small hairpin RNA

SLC1A5 - solute carrier family 1 member 5

SLC3A1 - solute carrier family 3 member 1

SLC7A5 - solute carrier family 7 member 5

SNAT1/2 - sodium-coupled neutral amino acid transporter 1

STAT - signal transducer and activator of transcription

SRC - spare respiratory capacity

STING - stimulator of interferon genes

TAM - tumor associated macrophages

TCA cycle - tricarboxylic acid cycle

TCR - T cell receptor

TG - triglyceride

TGF-β - transforming growth factor beta

Th - T helper cells

TIGAR - TP53 induced glycolysis regulatory phosphatase

TIGIT - T cell immunoglobulin and ITIM domain

TILs - tumor-infiltrating lymphocytes

TIM - T cell–immunoglobulin–mucin domain

TME - tumor microenvironment

TNF - tumor necrosis factor

Treg - regulatory T cells.
